# Structural and effective brain connectivity in focal epilepsy

**DOI:** 10.1016/j.ynirp.2025.100274

**Published:** 2025-06-27

**Authors:** S.B. Jelsma, M. Zijlmans, I.B. Heijink, F.W.A. Hoefnagels, M. Raemaekers, W.M. Otte, N.E.C. van Klink, D. van Blooijs

**Affiliations:** a Department of Neurology and Neurosurgery, University Medical Center Utrecht Brain Center, University Medical Center Utrecht, P.O. box 85500, 3508, GA, Utrecht, the Netherlands; b Stichting Epilepsie Instellingen Nederland (SEIN), the Netherlands; c Technical Medicine, University of Twente, Enschede, the Netherlands

**Keywords:** Diffusion-weighted imaging tractography, Intracranial EEG, Single pulse electrical stimulation, Network topology, Structural connectivity, Effective connectivity

## Abstract

Epilepsy surgery is usually based on the removal of a local epileptogenic zone. If epilepsy is considered a network disease, a network approach might be more suitable. Insight into patient-specific epileptic brain networks is necessary to establish network-based surgical strategies.

We included epilepsy surgery candidates who underwent diffusion-weighted imaging and intracranial EEG implantation with single pulse electrical stimulation (SPES, 0.2 Hz, 1–8 mA, 1 ms, monophasic stimuli) during presurgical evaluation. We reconstructed structural connectivity using fiber tractography taking intracranial electrodes as nodes. We reconstructed effective connectivity with SPES cortico-cortical evoked responses. We determined the inter-modal similarity between structural and effective connectivity with the Jaccard index, and compared network topologies using degree and betweenness centrality. We constructed a linear multilevel model to evaluate the relation between structural and effective connectivity at subject group level. The seizure onset zone nodes (SOZ), node proximity, and the volume of the electrode contact areas (VEA) were added to the model as possible predictors to accommodate for epilepsy and irregular spatial sampling.

We included 13 patients (five with electrocorticography, eight with stereo-EEG). The median Jaccard index was 0.25 (IQR: 0.20–0.29), which means there is a higher overlap than expected by chance (median expected Jaccard index = 0.1 (IQR: 0.07–0.17)) with a considerable amount of connections that did not overlap. The structural connectivity degree showed a significant positive correlation with the effective connectivity degree in 9/13 patients and at group level after accommodating for node proximity (β = 0.13, 95 %-CI = [0.04, 0.21], t(852) = 2.79, p = 0.0054). SOZ and VEA were no significant predictors for the correlation between structural and effective connectivity.

We showed a moderate overlap between non-invasive structural (measured with DWI) and invasive effective (measured with SPES) connectivity in epileptic brain networks. This overlap supports using non-invasively determined connectivity along with intracranial EEG to understand the epileptic brain. Future research needs to translate these findings towards network-based surgical strategies.

## Highlights

• DWI-based structural and invasive SPES-based effective connectivity show overlap.
• Moderate overlap supports use of non-invasive connectivity along with invasive EEG.
• Connectivity correlates independently from differences in spatial sampling.
• The seizure onset zone electrodes were not predictors in the linear multilevel model.
• Network-based surgical strategies need fusion of complementary connectivity analyses.

## Abbreviations:

SPES: = Single pulse electrical stimulation
DWI: = diffusion-weighted imaging
FT: = fiber tractography
iEEG: = intracranial EEG
sEEG: = stereo EEG
ECoG: = electrocorticogram
EZ: = epileptogenic zone
SOZ: = seizure onset zone
VEA: = volume of the electrode contact area
SC: = structural connectivity
EC: = effective connectivity

## 1 Introduction

Epilepsy surgery nowadays focuses on the removal of the epileptogenic zone (EZ). This spatially confined cortical area must be removed to achieve seizure freedom (Luders et al., 1993). The concept of focal epilepsy has evolved into a concept of an epileptogenic network with diseased nodes and connections related to the original EZ (van Blooijs et al., 2018), (Van Diessen et al., 2013a), (Slinger et al., 2022a), (Bartolomei et al., 2017), (Zijlmans et al., 2019), (Bui et al., 2015), (Novitskaya et al., 2023), (hao Guo et al., 2020), and with crucial nodes beyond the EZ in larger brain networks (Lopes et al., 2017), (Hebbink et al., 2017), (Slinger et al., 2022). With the focal approach to estimate the EZ, 25–50 % of the patients do not become seizure-free after surgery (Lamberink et al., 2020). A network approach focusing on diseased connections in the brain's network, rather than just the EZ, might improve the surgical outcome in some patients (Hebbink et al., 2017). This network approach is now purely an analytical framework and not yet based on a universally agreed-upon anatomical definition of the epileptogenic network. An anatomical definition of the epileptogenic network is difficult to define yet since a wide range of methodologies are used to describe networks (Slinger et al., 2022a). Intracranial EEG (iEEG) is currently used in clinical practice to determine the final surgical strategy in complex cases, but does not sample the whole brain. Whole-brain approaches to characterize brain networks could complement the iEEG, but the relationship between whole-brain and locally sampled network changes is poorly understood (Parker et al., 2018), (Crocker et al., 2021). In this study, we compare two approaches to characterize brain connectivity and investigate the influence of epilepsy on their relation.

We can characterize brain connectivity and identify connectivity alterations due to epilepsy with functional, effective, or structural connectivity analyses. Functional connectivity describes the temporal correlations between brain areas, without taking their directionality into account (Bartolomei et al., 2017). In this study, we focus on effective and structural connectivity as those modalities may complement each other in mapping the epileptic brain. Effective connectivity analyses describe the influence that brain areas directly exert over another by perturbating one area with for example single pulse electrical stimulation (SPES) on intracranial electrodes (Bartolomei et al., 2017) (Valentín et al., 2002). Studies using effective networks suggest that the epileptogenic network is densely interconnected (van Blooijs et al., 2018), (hao Guo et al., 2020), (Boido et al., 2014), (Boulogne et al., 2022). Structural connectivity analyses describe the anatomical connections between brain areas via white matter tracts and can be reconstructed with diffusion-weighted imaging (DWI) (Alger, 2012). Studies using structural networks suggest that the whole-brain structural networks in epilepsy patients are less efficiently organized (Slinger et al., 2022a), (Campos et al., 2015), (Jiang et al., 2017), (Bernhardt et al., 2019). Both structural and effective connectivity have their limitations which hampers the understanding of how epilepsy alters the complex brain network. Characterizing effective connectivity requires invasive implantation of electrodes which restricts the spatial sampling and resolution. Structural connectivity does not include directionality information.

Structural connectivity based on DWI non-invasively reveals biologically plausible neuronal pathways that may be followed by effective connectivity (Stam and van Straaten, 2012). Modeling of connectivity in the healthy, resting-state brain reveals that functional connectivity emerges from local neural network dynamics constrained by structural connections (Deco et al., 2013), (Cabral et al., 2017), (Xie et al., 2021). Therefore, there might be a large overlap between structural and effective connectivity. Two studies compared structural and effective (SPES-based) brain connectivity (Crocker et al., 2021b), (Parker et al., 2018b). Crocker et al. found a strong correlation between physiologically effective and structural connectivity networks (Crocker et al., 2021b). Parker et al. found a weak correlation (Parker et al., 2018b), indicating that effective and structural connectivity map distinct brain networks. Crocker et al. focused only on the physiological part of the network, while Parker et al. also included the suspected epileptogenic parts which may explain the large difference in the reported correlation. This discrepancy indicates the need to further elucidate the relation between structural and effective connectivity using a method that considers the influence of epilepsy.

We aimed to elucidate how structural and effective connectivity interrelate in the epileptic brain.

## 2 Methods

### 2.1 Patient cohort

We selected data from all people with drug-resistant focal epilepsy who underwent both a diffusion-weighted MRI and long-term, intracranial EEG (iEEG) monitoring between 2018 and 2021. Inclusion criteria were the presence of a DW image, a 3DT1-weighted MRI, a post-implantation CT scan, and a SPES procedure. Clinical data were extracted from the RESPect database which consists of iEEG data from epilepsy surgery candidates treated at the University Medical Center Utrecht, the Netherlands (Demuru et al., 2022). The Medical Ethical Committee of the UMC Utrecht approved collecting pseudo-anonymized data in the RESPect database. Written informed consent was obtained. 83 patients underwent iEEG monitoring and gave informed consent, of whom 13 also underwent a diffusion-weighted MRI.

### 2.2 Image acquisition

DWI was acquired up to a year pre-implementation with a multi-slice, multi-shot echo-planar imaging (EPI) sequence with an echo time (TE) of 91 ms, repetition time (TR) of 3191 ms, and an isotropic resolution of 2 mm. A total of 62 diffusion sensitizing gradient direction images with a b value of 1600 smm2 and a single $b_{0}$ scan were obtained**.** A 3D T1 with an isotropic resolution of 1 mm was acquired during the DWI scan for anatomical reference (‘MRI-DWI’). For processing of the electrode locations, we used a CT scan with an isotropic resolution of 1 mm after electrode implantation and another 3D T1 acquired before electrode implantation (‘MRI-CT’).

### 2.3 iEEG recording and SPES

The placement and type of the intracranial electrodes were determined clinically. iEEG was recorded with either electrocorticography (ECoG) or stereo electroencephalography (sEEG). The ECoG electrodes had an interelectrode contact distance of 1 cm and consisted of platinum electrode contacts with a 4.2 mm^2^ contact surface embedded in silicone (Ad-Tech, Racine, WI). The cylindrical sEEG electrodes were either platinum contacts with an 8.3 mm^2^ contact surface and 5 mm interelectrode contact distance (Ad-Tech, Racine, WI) or platinum/iridium contacts with a 5.0 mm^2^ contact surface and 3.5 mm interelectrode distance (DIXI Medical, France). iEEG data were recorded at 2048 Hz with a MicroMed LTM64/128 express EEG headbox with an integrated programmable stimulator (MicroMed, Mogliano—Veneto, Italy) and extracranial reference electrode on the scalp vertex or mastoid. We excluded electrodes not placed in the grey matter or recording noisy signals from further analysis. The treating neurophysiologist of each patient determined which electrode contacts were located on the SOZ independently of and prior to this study, noted in a dedicated section ‘for research’ of the clinical iEEG report. The Single Pulse Electrical Stimulation (SPES) protocol consisted of stimulus trials for each pair of adjacent electrode contacts. Each trial consisted of ten monophasic pulses with a pulse width of 1 ms, a current of 2 mA for sEEG and 8 mA for ECoG, and a repetition rate of 0.2 Hz. A lower current of 1 mA for sEEG or 4 mA for ECoG was used in electrodes placed in the hippocampus, amygdala, in the primary sensorimotor cortex, or in case of pain due to activation of dural c fibers or the trigeminal nerve.

### 2.4 Structural connectivity

The DWI was processed using the MRtrix3 package and MATLAB version R2021b (The Mathworks Inc., Natick, Massachusetts) (Tournier et al., 2019), combined with the brain imaging toolboxes Freesurfer, FSL, ANTs, and SPM12 (Smith et al., 2004), (Larsen et al., 2014), (Fischl, 2012), (Friston et al., 2006). The DWI data were denoised and corrected for common distortions as earlier described (Ades-Aron et al., 2018). Marchenko-Pastur Principal Component Analysis (MP-PCA) denoising (Veraart et al., 2016a), (Veraart et al., 2016b), Gibbs ringing correction (Kellner et al., 2016), EPI distortion correction (Holland et al., 2010), Eddy current correction (Andersson and Sotiropoulos, 2016), movement distortion correction (Pierpaoli et al.), b0 field inhomogeneity correction (Smith et al., 2004), (Andersson et al., 2003), and b1 bias field correction (Smith et al., 2004b), (Zhang et al., 2001) were performed. The MRI-DWI was co-registered linearly to the DWI $b_{0}$ image using the SPM12 toolbox.

**Fig. 1:**
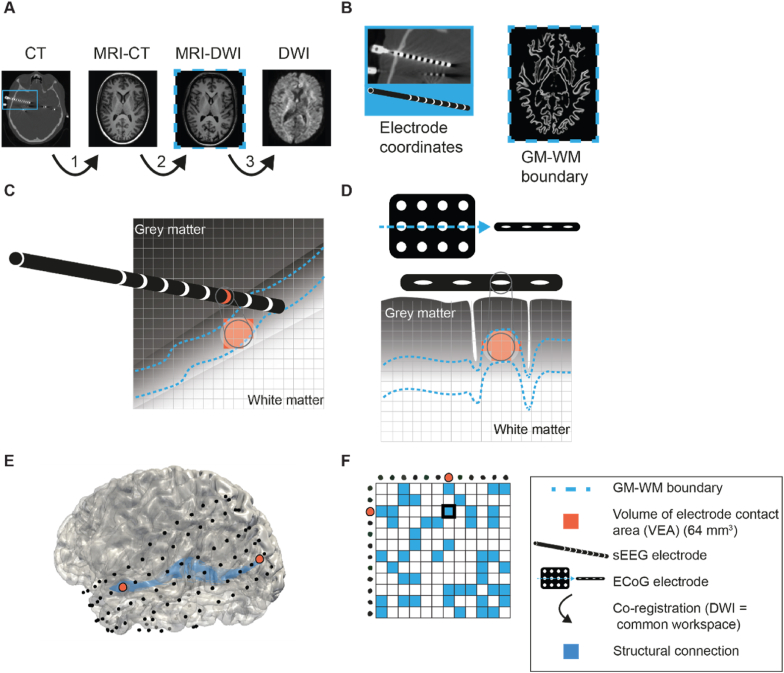
**Schematic overview of the structural connectivity network reconstruction based on co-registration, electrode contact area definition, and fiber tractography. A)** The common workspace is defined by the DWI via: 1) Linear co-registration of the post-implantation CT with the MRI-CT. 2) Linear co-registration of the MRI-CT with the MRI-DWI. 3) Linear co-registration of the MRI-DWI with the DWI. **B)** Electrode contact coordinates were extracted from the CT. The grey-white matter boundary was segmented from the MRI-DWI. **C)** The electrode contact coordinate from the sEEG was projected on the grey-white matter boundary and the closest 64 voxels were used to create the electrode contact area. **D)** The same procedure as in C) was followed for ECoG. **E)** Structural connectivity was reconstructed by fiber tracking the white matter tracts between electrode contact areas. **F)** Binary structural connectivity networks were made with each node representing an electrode contact area. A structural connection (blue boxes) was formed when the streamline density exceeded a threshold of 0.1. The connection in E) is highlighted with orange colored electrodes and a thick border around the blue box. sEEG = stereo EEG, ECoG = electrocorticogram, DWI = diffusion-weighted imaging, MRI-CT = MRI prior to electrode implantation, which was used to co-register the CT after electrode implantation MRI-DWI = MRI acquired at the time of the DWI scan. GM-WM boundary = grey matter – white matter boundary.

We defined the common workspace by the DWI. The post-implantation CT scan was co-registered linearly to the MRI-CT and intracranial electrode contact coordinates were extracted from the CT scan (Fig. 1). We co-registered the MRI-CT to the MRI-DWI. The inverse transformation matrix was used to transform the electrode contact coordinates to the space of the MRI-DWI. The MRI-DWI was segmented in binary brain masks including grey and white matter. The electrode contact coordinates were projected onto the grey-white matter boundary mask to create electrode contact areas, containing grey-white matter voxels closest to each coordinate. A maximum of the closest 64 grey-white matter voxels were assigned to each electrode contact area. This volume of the electrode contact area (VEA) of max 64 mm^3^ is comparable to the estimated size of the locally activated brain area with SPES (Silverstein et al., 2020). Overlapping voxels, closest to more than one projected electrode contact, were assigned to only the nearest electrode contact, which resulted in some contact areas having a VEA of less than 64 mm^3^. The electrode contact areas were used as seed and termination regions for fiber tractography.

Structural connectivity was reconstructed by fiber tracking the white matter tracts between electrode contact areas. Anatomical-constrained probabilistic tractography (ACT) (Smith et al., 2012) was performed using the iFOD2 reconstruction algorithm (Tournier et al., 2010) with a seed density of 6000 seeds per voxel and constraints on: the fiber orientation distribution (FOD) threshold = 0.15, maximal angle = 70°, minimal streamline length = 4 mm, and maximal streamline length = 400 mm. The response function for constrained spherical deconvolution (CSD) was estimated from the DWI data with the Dhollander algorithm (Dhollander et al., 2019). The FOD was calculated with the Multi-Shell Multi-Tissue CSD (MSMT-CSD) algorithm using two-tissue CSD (Tournier et al., 2012).

We created binary structural connectivity networks with each node representing an electrode contact area. All streamlines seeded inside an electrode contact area and terminating inside another electrode contact area were considered. The streamline density is the number of streamlines divided by the volume of the two involved electrode contact areas. A structural connection was formed when the streamline density exceeded a threshold of 0.1.

### 2.5 Effective connectivity

The ten stimuli of each SPES trial were epoched in time windows 2 s prior to 2 s post stimulus, time-locked to the stimulus artifact. We re-referenced the epochs in the referential montage subtracting the median of the 10 % signals with the lowest variance in the same trial. These ten epochs for each trial were averaged per electrode contact and the baseline median of the signal during 2 s pre-stimulation was subtracted. The obtained post-stimulus signal is the evoked response potential (ERP). Cortico-cortical evoked potentials (CCEPs) were detected in this ERP using an automatic detector (van Blooijs et al., 2018). The detector was optimized for CCEP detection compared to human reviewers for ECoG and sEEG signals separately. For ECoG data, ERPs were classified as CCEP if they occurred within 9–100 ms after stimulation, had a negative sharp potential, and their amplitude exceeded 2.6 times the standard deviation (SD) of the baseline before stimulation (van Blooijs, 2015). For sEEG data, the ERP had to exceed 3.5 times the baseline SD and could have a negative or a positive early sharp potential (Jelsma, 2022).

**Fig. 2:**
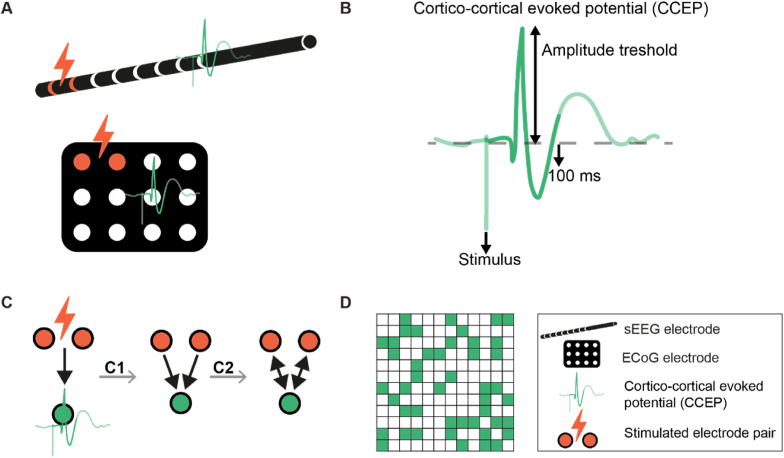
**Schematic overview of the effective connectivity network reconstruction based on single pulse electrical stimulation, detection of cortico-cortical evoked potentials, and bi-directional network definition. A)** The SPES protocol consisted of stimulus trials for each pair of adjacent electrode contacts. Each trial consisted of ten monophasic pulses with a pulse width of 1 ms, a current of 1–2 mA for sEEG and 4–8 mA for ECoG, and a repetition rate of 0.2 Hz. **B)** For ECoG data, CCEPs were detected if they occurred within 9–100 ms after stimulation, had a negative sharp potential, and their amplitude exceeded 2.6 times the standard deviation (SD) of the baseline before stimulation. For sEEG data, the CCEP had to exceed 3.5 times the baseline SD and could have a negative or a positive early sharp potential**. C1)** Connections were drawn from both electrode contacts in a stimulus pair to the electrode contacts in which a CCEP was detected. **C2)** The connections were made bi-directional to allow comparison to structural connectivity. **D)** Binary effective connectivity networks were made with each node representing an electrode contact. sEEG = stereo EEG, ECoG = electrocorticogram, SPES = Single pulse electrical stimulation, CCEP = cortico-cortical evoked potentials, ERP = evoked response potential.

We created binary effective connectivity networks with each node representing an electrode contact (Fig. 2). Connections were drawn from both electrode contacts in a stimulus pair to the electrode contacts in which a CCEP was detected. Effective connectivity networks were made symmetrical by considering all connections derived from CCEPs as bi-directional. This bi-directional assumption makes the effective connectivity networks lose their unique directional component as opposed to functional or structural connectivity, but is necessary to fairly compare them to structural connectivity.

### 2.6 Inter-modal similarity

The inter-modal similarity between structural and effective connectivity networks was determined with the Jaccard Index (JI) calculated as the size of the set of intersecting connections divided by the size of the set of union connections:

(1.1) $$\text{Jaccard}\;\text{Index}\;\left(JI\right)=\frac{SC⋂EC}{SC⋃EC}$$

with SC and EC the structural and effective connectivity matrixes, and a JI between 0 (no overlap) and 1 (perfect overlap). The Jaccard/Tanimoto similarity hypothesis test was conducted, with the R package ‘Jaccard’, to determine significance if the JI was higher than expected by chance given the densities of the structural and effective connectivity (Chung et al., 2019). The expected JI is the JI when the connections are placed at random positions in the connectivity network, calculated with:

(1.2) $${JI}_{\text{expected}}=\frac{d_{SC}d_{EC}}{d_{SC}+d_{EC}-d_{SC}d_{EC}}$$

with $d_{SC}$ and $d_{EC}$ the densities of the structural and effective connectivity matrices SC and EC.

The density is defined as:

(1.3) $$d_{SC}=\frac{\sum SC}{N*\left(N-1\right)}$$

with SC the structural (or effective) connectivity matrix, and N the number of electrode contacts. The ratio between structural and effective connections in the set of symmetric difference connections was calculated with:

(1.4) $${Ratio}_{SC\Delta EC}=\log_{10}\left(\frac{SC-\left(SC⋂EC\right)}{EC-\left(SC⋂EC\right)}\right)$$

with SC and EC the structural and effective connectivity matrixes, and a ratio between 0 (equal amount of structural and effective connections) and ±∞ (zero structural or zero effective connections).

### 2.7 Network topology

The topology of the structural and effective connectivity was determined with the degree and betweenness centrality. Each network characteristic was calculated per node, thus electrode contact (area). The degree was calculated with:

(1.5) $$Degree\;\left(j\right)=\sum_{j=1}^{n}A_{ij}$$

with n the number of nodes in the connectivity network, $A_{ij}$ the presence (1) or absence (0) of a connection between the i th and j th node. The betweenness centrality was calculated with:

(1.6) $$Betweenness\;centrality\;\left(v\right)=\sum_{s\neq v\neq t}\frac{\sigma_{st}\left(v\right)}{\sigma_{st}}$$

with $\sigma_{st}\left(v\right)$ the number of shortest paths from node s to node t that pass through node v, and $\sigma_{st}$ the total number of shortest paths from node s to node t.

**Fig. 3:**
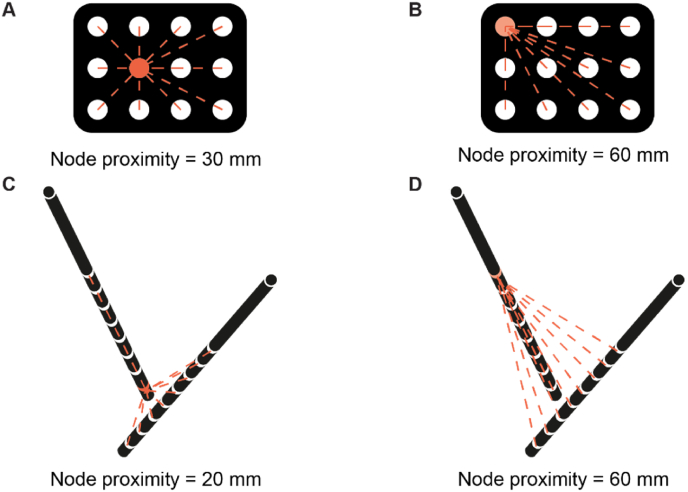
**The node proximity, the distance between one node and all other nodes, computed as the median Euclidean distance between electrode contact coordinates. A)** Node from ECoG with a low node proximity. **B)** Node from ECoG with a high node proximity. **C)** Node from sEEG with a low node proximity. **D)** Node from sEEG with a high node proximity. sEEG = stereo EEG, ECoG = electrocorticogram.

We used Spearman's ρ test to calculate the correlation between structural and effective connectivity for the degree and betweenness centrality at patient level. The network characteristics that showed a significant correlation were further assessed at group level with linear multilevel analyses. We used the network characteristic of the structural connectivity as dependent variable. We first fitted an intercept-only model to quantify the dependency in the data with the intraclass correlation (ICC). The ICC was calculated as:

(1.7) $$ICC=\frac{\sigma_{inter}^{2}}{\sigma_{intra}^{2}+\sigma_{inter}^{2}}$$

with $\sigma_{inter}^{2}$ and $\sigma_{intra}^{2}$ as the inter- and intra-data variance, and an ICC between 0 (no variation between patients) and 1 (no variation within patients). We constructed a linear multilevel model with backward elimination of predictors with the R package ‘lme4’. The possible predictors were the node proximity (the distance between one node and all other nodes, see Fig. 3), the volume of the electrode contact areas (VEA, see Fig. 1), and the SOZ nodes (electrodes in the SOZ as determined by the clinicians, see above). The node proximity was the median distance between a node and all other nodes computed as the median Euclidean distance between electrode contact coordinates. We included the node proximity in the model to account for the impact of the irregular spatial sampling of the brain with sEEG or ECoG. The presumed SOZ is extensively sampled in sEEG and often located in the middle of the ECoG, thus has a low node proximity, which results in a higher probability of a high degree or betweenness centrality (van Blooijs et al., 2018). Therefore, correcting for node proximity is mandatory to make valid conclusions about the influence of epilepsy (Crocker et al., 2021). Statistical analyses were performed in R 4.1.2 (R Core Team, 2022). We corrected for multiple testing with FDR-correction (p < 0.05) (Benjamini and Hochberg, 1995).

## 3 Results

### 3.1 Patient characteristics

Thirteen patients met the inclusion criteria: eight patients underwent sEEG and five patients underwent ECoG (Table 1). The median age was 25y (range: 10-50y), five patients were younger than 18y. Four patients were seizure free (ILEA 1) after epilepsy surgery, three patients did not have a resection after the intracranial monitoring period.

**Table 1:** **Patient characteristics** In two patients (9,13) the SOZ could not be determined due to diffuse seizure onset. In one patient (3) the SOZ is not resected due to overlap of the SOZ with functional area. Electrodes were included when placed in the grey matter, excluding the electrodes with noisy signals. The outcome is determined with the ILAE classification. sEEG = stereo EEG, ECoG = electrocorticogram, iEEG = intracranial EEG, f = female, m = male, F = frontal; T = temporal; P = parietal; C = pre or post-central gyrus; O = occipital; IH = interhemispheric; A = amygdala; H = hippocampus; I = insula R = right; L = left, SOZ = seizure onset zone, ND = not determined; mMCD = mild malformation of cortical development; FCD = focal cortical dysplasia; NP = no pathology found; NR = not resected.

Patient	ECoG/sEEG	Age at iEEG	Sex	iEEG location	Sampled hemisphere	Included electrodes (all electrodes)	Electrodes in SOZ	Outcome (ILAE) (follow up (months))	Pathology
1	ECoG	15	f	T,P,O	L	104 (112)	37	5 (13)	NP
2	ECoG	28	f	T	L	71 (72)	4	5 (25)	mMCD
3	ECoG	37	m	F	L	61 (64)	4	NR	NR
4	ECoG	44	m	F,T,IH	L	67 (80)	15	1 (7)	FCD type 2A
5	ECoG	18	m	F,T	L	58 (64)	11	3 (17)	mMCD
6	sEEG	45	f	F,T,A,H	R	44 (67)	14	1 (30)	DNET grade 1
7	sEEG	50	f	F,T,A,H	R & L	47 (88)	4	1 (27)	NP
8	sEEG	50	f	T,P,A,H	R	50 (78)	9	2 (7)	Gliosis
9	sEEG	25	m	F,T	R & L	101 (121)	ND	NR	NR
10	sEEG	17	f	T,P,O,A,H	R	68 (90)	21	2 (10)	mMCD
11	sEEG	17	f	F,C,T,P,I	L	47 (89)	11	1 (7)	mMCD
12	sEEG	14	m	F,T,A,H,I	L	58 (103)	4	5 (13)	mMCD
13	sEEG	10	f	F,T,A,H,I	L	81 (142)	ND	NR	NR

### 3.2 Inter-modal similarity

**Fig. 4:**
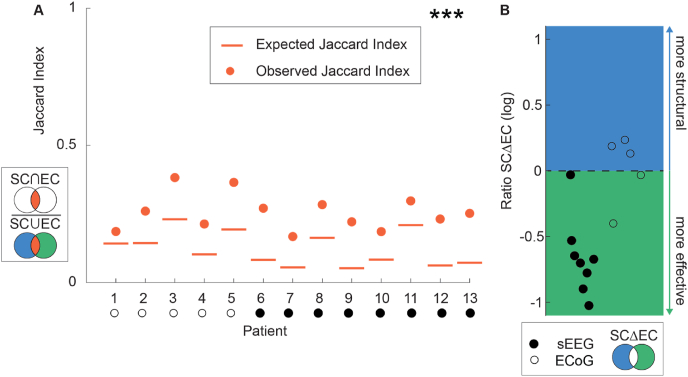
**Inter-modal similarity as determined by the Jaccard index and the set of difference connections A)** The inter-modal similarity determined by the JI was for all patients significantly higher than the expected JI. We corrected for multiple testing with FDR-correction (p < 0.05). **B)** A ratio between structural and effective connections of −1 indicates 10 times more effective connections. In the set of difference connections, all sEEG patients had more effective connections. For ECoG patients the ratio between structural and effective connections differed per patient. JI = Jaccard Index SCΔEC = set of difference connections, ${Ratio}_{SC\Delta EC}=\log_{10}\left(\frac{SC-\left(SC⋂EC\right)}{EC-\left(SC⋂EC\right)}\right)$, sEEG = stereo EEG, ECoG = electrocorticography.

**Fig. 5:**
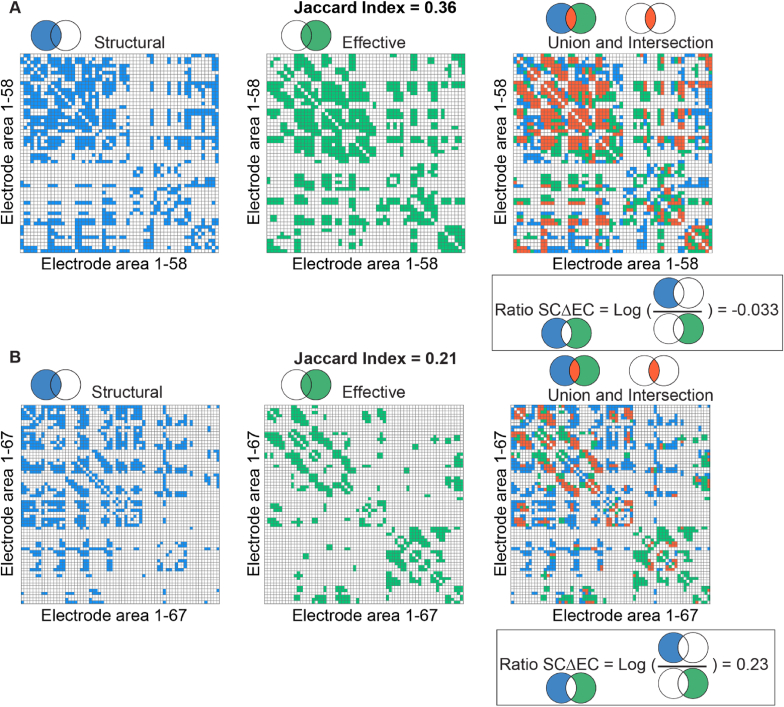
**Example of structural, effective, and overlapping connectivity matrixes of two patients** Left: structural connectivity matrix in blue. Middle: effective connectivity matrix in green. Right: Union (blue & green) and Intersection (orange) of structural and effective connectivity matrixes. **A**) ECoG patient 5 with a JI of 0.36. In the right figure, we observe the overlapping and union connections. The Jaccard Index indicates many effective connections that overlap with structural connections (orange). The ratio indicates that the number of only effective connections (green) is similar to that of only structural connections (blue). **B**) ECoG patient 4 with a JI of 0.21, indicating that a small number of connections are present in both structural and effective connectivity. The ratio indicates that the number of only structural connections (blue) is larger than the number of only effective connections. SCΔEC = set of difference connections, ${Ratio}_{SC\Delta EC}=\log_{10}\left(\frac{SC-\left(SC⋂EC\right)}{EC-\left(SC⋂EC\right)}\right)$.

The median inter-modal similarity, measured with the JI, between structural and effective connectivity, was 0.25 (interquartile range (IQR) = 0.20–0.29). The JI was higher (p < 0.0001) than expected by chance given the densities of the networks in all patients (Fig. 4A). In the set of difference connections, the ratio between structural and effective connections showed more effective connections in all sEEG patients (Fig. 4B). An example of structural and effective connectivity matrices of two ECoG patients are shown in Fig. 5.

### 3.3 Network topology

**Fig. 6:**
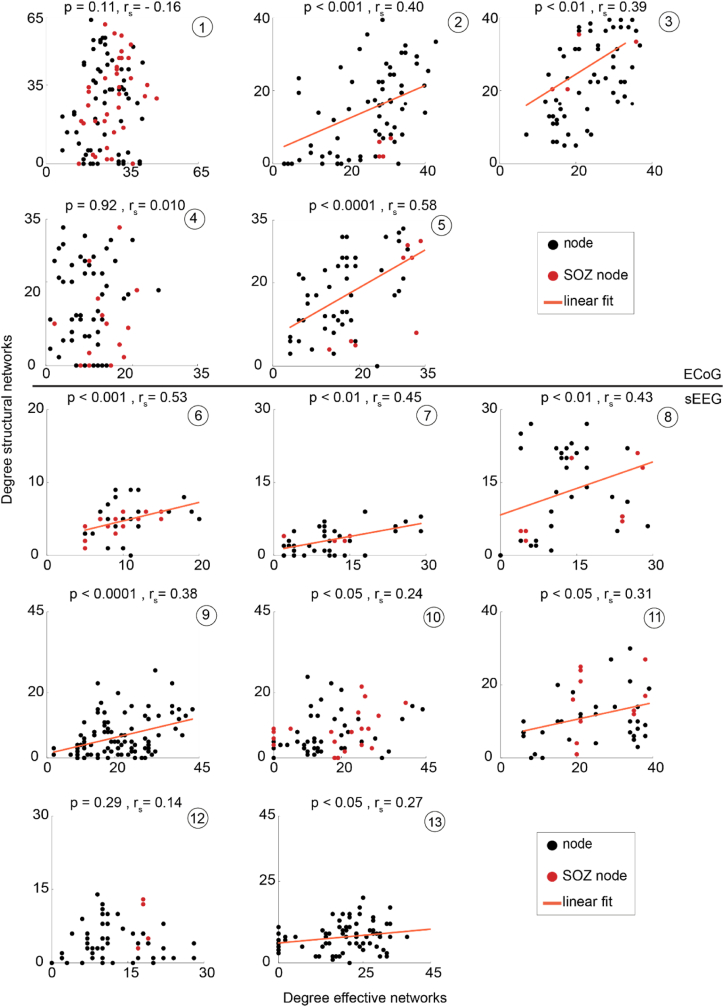
**Correlation between the degree of effective and structural networks.** In 9/13 patients the structural connectivity degree was positively correlated to the effective connectivity degree. The orange lines are the best linear fit through the data points. The red dots indicate the seizure onset zone (SOZ) nodes. In patient 9 and 13, the SOZ was not determined. We corrected for multiple testing with FDR-correction (p < 0.05). SOZ = seizure onset zone, $r_{s}$ = correlation coefficient spearman's ρ test, sEEG = stereo EEG, ECoG = electrocorticography.

We observed a positive correlation between effective connectivity degree and structural connectivity degree in 9/13 patients (Fig. 6). We found a correlation between betweenness centrality of effective connectivity and structural connectivity for four patients. After FDR correction, three patients remained significant (3/13). We did not further assess this correlation at group level with linear multilevel analyses (Fig. S5).

**Fig. 7:**
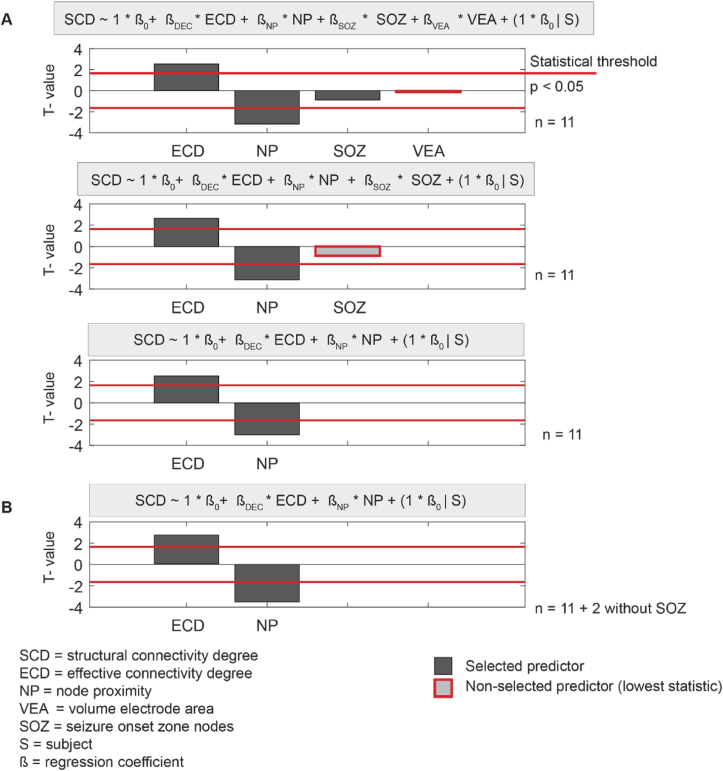
**The characteristics of the fitted linear multilevel models.** The linear multilevel model showed that the node proximity and the effective connectivity degree were associated with the structural connectivity degree at a group level. We evaluated the effective network characteristic, the node proximity, the volume of the structural electrode contact areas, and the seizure onset zone (SOZ) nodes as possible predictors in the model. The T-value is the regression coefficient β divided by its standard error. **A)** All steps of the backward elimination are shown: step one includes VEA, SOZ, and NP, step two includes SOZ and NP and step three includes ECD and NP. We included the eleven patients with a defined SOZ in these models. What can be seen is that the final model includes the effective connectivity degree and the node proximity, with respectively a positive and negative correlation. The volume of electrode areas (VEA) and SOZ nodes were not associated with the structural connectivity degree. **B)** The final model with all 13 patients included. What can be seen is that the structural connectivity degree is positively correlated to the effective connectivity degree (t(852) = 2.79) and negatively correlated to the node proximity (t(852) = −3.47). SCD = structural connectivity degree, ECD = effective connectivity degree, NP = node proximity, VEA = volume electrode area, SOZ = seizure onset zone nodes, S = subject, β = regression coefficient.

The effective connectivity degree and the node proximity were associated with the structural connectivity degree (Fig. 7). We included the eleven patients with a delineated SOZ for the models that included the SOZ nodes. The ICC was 0.37, which indicates that the inter-patient variance caused a considerable amount of the variance in the data. The effective connectivity degree was positively correlated to the structural connectivity degree after accommodating for node proximity with a regression coefficient (β) of 0.13, 95 %-Confidence Interval (CI) [0.04, 0.21], t(852) = 2.79, p = 0.0054, marginal R^2^ = 0.04, conditional R^2^ = 0.42. The volume of electrode areas (VEA) and SOZ nodes did not significantly influence this correlation nor were they correlated themselves to the structural connectivity degree (see Fig. S4). The node proximity was negatively correlated to the structural connectivity degree with a regression coefficient (β) of −0.13, 95 %-CI [−0.21, −0.06], t(852) = −3.47, p < 0.0001, in concordance with Fig. S2.

## 4 Discussion

We compared structural and effective connectivity in 13 patients with epilepsy. We found a moderate inter-modal similarity between structural and effective connectivity with a JI of 0.25 (IQR: 0.20–0.29). The structural connectivity degree compared to the effective connectivity degree showed a positive correlation in 9/13 patients, indicating a similar network topology. We did not find a consistent correlation between the betweenness centrality of effective connectivity and the betweenness centrality of structural connectivity at patient level. After correcting for the inter-patient variance and the bias caused by the node proximity with multilevel modeling, there was a correlation between the structural and effective connectivity degree at group level. The node proximity and effective connectivity degree were significant predictors in the multilevel model.

Earlier work that integrated effective and structural connectivity concluded that there is a significant correlation between the two modalities (Parker et al., 2018), (Crocker et al., 2021), (Silverstein et al., 2020), (Conner et al., 2011). Most of the studies (Crocker et al., 2021), (Silverstein et al., 2020), (Conner et al., 2011), focused on the correlation between properties of the overlapping connections, while we focused on all connections in both connectivity networks.

Our findings regarding the inter-modal similarity are in concordance with Parker et al. They reported a median JI of 0.17 (IQR: 0.16–0.20), with a median expected JI of 0.04 (IQR: 0.04–0.08) (Parker et al., 2018b). This lower JI compared to our research can be explained by lower densities of both the structural (0.09 vs 0.20) and effective (0.10 vs 0.23) connectivity networks as the JI is directly dependent on the density of the network. Possible explanations are differences in sampling location since Parker et al. did not sample the temporal lobe, differences in SPES and DWI acquisition, or distinct choices in connectivity reconstruction. Although the JI was higher than expected by chance given the densities of the networks, there were still many structural and effective connections that did not overlap. Especially sEEG patients showed more effective connections than structural connections. For ECoG patients, this differed per patient. A difference in comparing structural and effective connectivity was expected between sEEG and ECoG, because they act on different spatial sampling scales and anatomical locations resulting in distinct network properties (Yaffe et al., 2015), (Bernabei et al., 2021). sEEG can sample the whole brain, including the deep sulci and left and right hemispheres, with a lower local 3D spatial resolution. In contrast, ECoG samples a broad area of the outer cortical surface with a high local spatial resolution. For structural connectivity, the brain area sampled with sEEG contains long-range white matter tracts from the right to the left hemisphere and more fiber crossing regions compared to ECoG. The errors in reconstructing these long white matter tracts and tracts in fiber crossing regions are generally bigger than short tracts in single-fiber regions, as seen in the outer cortical surface sampled by ECoG (Neher et al., 2015). For effective connectivity, the difference in the contact surface, contact shape, and interelectrode contact distance in sEEG required a different stimulation protocol and subsequently, we needed a different threshold to detect the CCEPs. This distinct CCEP detection protocol could be a reason why we observed more effective connections than structural connections in sEEG compared to ECoG patients.

A correlation is expected between the topology of the structural and effective connectivity since we hypothesized that they both map the patient's brain network (Stam and van Straaten, 2012), (Matsumoto et al., 2012). We did find a significant correlation between the structural and effective connectivity degree in 9/13 patients. For four patients, ECoG patients 1, 4, and sEEG patients 10, 12, we did not find a correlation between the structural and effective connectivity degree. In patient 4, the focal cortical dysplasia possibly altered the grey-white matter boundary which may have disturbed the seeding of streamlines in that area. The iFOD2 reconstruction algorithm probably performs differently in focal epilepsy patients with large white matter abnormalities or structural lesions affecting the grey-white matter boundary. It can therefore be useful in describing the deviated structural pathways around those lesions (Campos et al., 2015). In patients 10 and 12, mild malformations of cortical development were found; in patient 1, no structural abnormalities were found in pathology.

Previous studies have observed alterations in the degree around the SOZ in structural and effective connectivity (Parker et al., 2018), (Boido et al., 2014), (Guye et al., 2003). Parker et al. reported a higher effective connectivity outdegree in SOZ compared to non-SOZ nodes and a higher distribution of the structural connectivity degree within the SOZ (Parker et al., 2018). Boido et al. and Van Blooijs et al. reported a higher effective connectivity degree in the SOZ compared to non-SOZ nodes (Boido et al., 2014), (van Blooijs et al., 2018b). The SOZ was not a significant predictor in the multilevel model that described the correlation between the structural and effective connectivity degree, suggesting that network alterations due to epilepsy are similar or not present in structural and effective connectivity. Nine patients had a bad outcome (ILEA 2 or higher), thus delineation of the SOZ may be inaccurate and may have introduced noise to our multilevel model. The iEEG sampled only the brain areas that were suspected of epilepsy based on the pre-surgical evaluation. In the patients where no SOZ could be defined or with bad outcomes, one could argue that most of the network we sampled is epileptogenic network. Especially in the light of studies that reported altered connectivity beyond the SOZ in people with focal epilepsy compared to healthy controls (Barrios-Martinez et al., 2024), (Rigoni et al., 2024), (Duma et al., 2024), for example in important subcortical structures and mesial structures (Barrios-Martinez et al., 2024) or ipsilateral and contralateral to the temporal lobe SOZ (Rigoni et al., 2024).

The exact relation between structural and effective connectivity remains a complex question. Theoretically, effective connections describe the physiological organization of communication between brain areas. Structural networks could be seen as the supporting hardware that allows this communication. This assumption does not fully explain the relation between structural and effective connectivity. The structural connections could be non-functional, or communication could go via other ways than the white matter pathways from which the structural connections are inferred, for example, via cell-to-cell communication spreading over the cortical grey matter (Badawy et al.). Even when the communication transmits via the white matter pathways, ephaptic coupling between adjacent axons can result in communication flows via the extracellular space that is not reconstructed by structural fiber tracking (Sheheitli and Jirsa, 2020). Some studies hypothesize that especially in epileptogenic network parts, the structure-function coupling is disrupted (Parker et al., 2018b),(Van Diessen et al., 2013), (Stacey et al., 2015), (Sinha et al., 2023), (Duma et al., 2024). A median of 18 % [IQR = 7–25 %] of the included nodes were SOZ nodes, and probably even more nodes contained epilepsy (Bartolomei et al., 2017), (Barrios-Martinez et al., 2024). This may explain the moderate correlations we reported and indicate that structural and effective connectivity may be interchangeably used but also have the potential to complement each other. Duma et al. found stronger structure-function coupling in regions where seizures propagate and this effect might be increased with disease severity (Duma et al., 2024). In this study we did not find network alterations or structure-effective correlations related to epilepsy.

### 4.1 Strengths and limitations

A strength of this study is that we used multilevel modeling to elucidate the relation between structural and effective networks which allowed us to correct for node proximity, consider the influence of epilepsy, and analyze at group level. We used a heterogeneous group of patients in terms of age, pathology, seizure control after surgery, iEEG electrode type, and iEEG location which allowed us to generalize results of effective and structural connectivity among several patients. The ICC was 0.37, indicating a large variation among patients and showed that a multilevel model was necessary to analyze the correlation between the structural and effective connectivity degree at group level. One limitation is the small sample size which made it not possible to control for the covariates leading to the large variation among patients such as SOZ localizations and seizure control. While our current sample size provided sufficient power to detect the association between effective and structural connectivity, post-hoc power analyses indicated that substantially larger samples (N > 150) would be required to reliably assess the effect of seizure onset zone. Therefore, non-significant effects should be interpreted with caution. In the model, the node proximity was negatively associated with the structural connectivity degree. Independently, the node proximity showed a significant negative correlation with the degree of the node in both the structural and effective connectivity network (Supplementary material; Figs. S1 and S2). This agrees with Crocker et al., who reported a negative correlation between the node proximity, the amplitude of CCEPs, and the streamline density (Crocker et al., 2021).

The size of the connectivity networks in our dataset varied due to the clinically determined, patient specific iEEG configurations. Network size is known to affect network topology (van Wijk et al., 2010). Therefore, we focused on comparing structural and effective connectivity within patients. At group level, the multilevel modeling took account of the inter-patient difference in network size.

For the structural networks, we used a liberal threshold on the streamline density of 0.1, similar to Parker et al. (2018a). (Smith et al., 2015). Because of a lack of a standardised threshold, we optimized the streamline density threshold based on visual inspection of all connections. The choice of threshold influences the ratio of false negative and false positive connections (Sinke et al., 2018). Both false positive and negative structural connections are primarily due to the fiber tracking algorithm. Erroneous false positive structural connections are mainly formed in connections of shorter distances (Smith et al., 2015). Erroneous false negative structural connections are mainly formed in longer distances with large curvatures, and in region of crossing fibers (Smith et al., 2015). We decided to prioritize connectivity completeness. This resulted in potential inclusion of more erroneous structural connections which increases inter-subject variability and network density. Zalesky et al. recommends keeping the connection density lower than the ground truth for optimal network topology analyses (Zalesky et al., 2016). Due to the patient-specific iEEG configurations, we could not guess a ground truth network density but the density of the structural connectivity was lower than the density of the effective connectivity (0.20 vs 0.23).

Some researchers favor weighted network measures because they can hold complementary aspects of network organization (Rubinov and Sporns, 2010). Furthermore, binarization results in loss of information about connection strength. We deemed it necessary to dichotomize the structural network because the biological accuracy of the streamline density is limited when filtering of the streamlines to reduce inherent biases associated with fiber reconstruction is not performed (Smith et al., 2015). We did not perform this filtering due to a lack of a standardised filtering method for region-of-interest based fiber tracking without whole-brain coverage.

The choice of a referential montage in the CCEP analysis may have influenced the CCEP detection. A bipolar or other local montage has the advantage of high signal-to-noise ratio and higher spatial resolution (Mitsuhashi et al., 2020), but has the limitation of cancelation of CCEPs distributed over multiple neighboring electrodes (Huang et al., 2024). In the common average reference, high amplitude CCEPs might be contained and redistributed into all other channels (Huang et al., 2025). We used an extracranial referential montage and re-referenced with low-variance intracranial signals to improve the signal-to-noise ratio but mitigate the limitations of the common average reference. We did not find a consistent correlation for the betweenness centrality between structural and effective connectivity (Supplementary material; Fig. S5). The betweenness centrality is an advanced calculation to quantify hub nodes in a connectivity network. In normal brain networks, only a few nodes have a high betweenness centrality (Van Diessen et al., 2013a), (Stam and van Straaten, 2012). This intrinsic property of the betweenness centrality makes it sensitive to the noise caused by imperfections in estimating electrode contact areas. To determine the electrode contact areas used, we assumed the size of the local activated grey-white matter boundary with SPES (Supplementary material; Fig. S3). Due to the location of the electrodes relative to the grey-white matter boundary, some electrode contact areas overlapped. Overlapping voxels were assigned to the nearest electrode contact, which resulted in some contact areas having a volume of less than 64 mm^3^. We used the volume of the electrode contact areas (VEA) as a possible predictor in the multilevel model and did not find a significant correlation with the degree of the structural networks (Supplementary material; Fig. S4). Furthermore, the electrode orientation to the grey-white matter boundary modulates the CCEP which could have resulted in the highest contribution to the CCEP caused by an area of the grey-white matter boundary further away than our estimated electrode contact area (Paulk et al., 2022). This could explain the inconsistent correlation for the betweenness centrality we reported and indicates that this type of network measure requires knowledge about the whole brain network.

### 4.2 Future perspectives

The moderate overlap between structural and effective connectivity (JI = 0.25) shows that both structural and effective connectivity may be needed to guide network-based surgical strategies. We reason that both structural and effective connectivity are able to reveal the connections that have an average distance and follow large, single-fiber tracts. At the same time, structural and effective connectivity may complement each other by increasing the sensitivity of large distance connections and the specificity of short distance connections. Future research is needed into the not-overlapping connections and the importance of them in the ground truth network topology.

An integration of techniques is often used in epilepsy surgery to estimate the EZ and decide if surgery is feasible (Mouthaan et al., 2016). This study illustrates with only a moderate correlation between structural and effective connectivity that an integration of techniques is needed to determine an (epileptogenic) brain network in people with complex focal epilepsy. The epileptogenic network is defined by Bartolomei et al. as the brain regions involved in the production and propagation of epileptic activities and the connections between those regions (Bartolomei et al., 2017). Since it is hard to surgically remove a whole network, specific nodes and connections critical for seizure generation should be identified. We propose the term ‘epileptogenic network nexus’ as the theoretical equivalent of the epileptogenic zone. The nexus is the crucial hub node or connection that, when removed, leads to the disruption of the epileptogenic network and thus seizure freedom. The techniques to define this epileptogenic network nexus include but are not limited to structural and effective connectivity. Whole-brain scale structural connectivity could be evaluated with DWI to non-invasively identify important hub nodes and connections in the epileptogenic network for the surgical planning of intracranial electrodes. This first hypothesis of the epileptogenic network nexus could then be confirmed during iEEG monitoring with SPES-based effective connectivity and analysis of seizure propagation. In this study, we were not able to distinguish this epileptogenic network nexus but both structural and effective connectivity have shown to disclose (parts of) the epileptogenic network previously (van Blooijs et al., 2018), (Boido et al., 2014), (Barrios-Martinez et al., 2024). A limitation of iEEG studies is the lack of a control group. A strategy to further disclose the epileptogenic network nexus could be to define physiological population-averaged networks to distinguish abnormal structural-effective correlations in individuals. Development of such an atlas based on iEEG electrodes needs a large sample of ideally seizure-free patients to remove and average out an epileptogenic network. We did not include functional connectivity due to the many possible functional connectivity measures that also dynamically change over brain state (Bartolomei et al., 2017). Extensive efforts are made to elucidate the functional connectivity alterations due to epilepsy. The results of these analyses are highly variable (Slinger et al., 2022a). Functional connectivity networks based on EEG, iEEG, functional magnetic resonance imaging, or magnetoencephalography might be a useful addition in a future study, because they act on different temporal and spatial scales than effective and structural connectivity (Bullmore and Sporns, 2009). Quantifying the relation between structural, effective, and functional connectivity is important for integrating these networks to estimate the epileptogenic network nexus.

## 5 Conclusion

We explored the relation between structural and effective patient-specific epileptic brain connectivity. We conclude that structural and effective connectivity show a moderate inter-modal similarity and the structural and effective connectivity topology described by the degree correlates independently from common sources of bias.

## CRediT authorship contribution statement

**S.B. Jelsma:** Writing – original draft, Visualization, Software, Project administration, Methodology, Investigation, Formal analysis, Conceptualization. **M. Zijlmans:** Writing – review & editing, Supervision, Resources, Project administration. **I.B. Heijink:** Writing – review & editing. **F.W.A. Hoefnagels:** Writing – review & editing. **M. Raemaekers:** Supervision, Data curation. **W.M. Otte:** Writing – review & editing, Methodology. **N.E.C. van Klink:** Writing – review & editing, Validation, Supervision, Project administration, Methodology, Investigation, Data curation, Conceptualization. **D. van Blooijs:** Writing – review & editing, Validation, Supervision, Software, Project administration, Methodology, Investigation, Data curation, Conceptualization.

## Funding

10.13039/100015870DB, 10.13039/100030960SJ were supported by the Epilepsie-NL grant number 23-06.

MZ was supported by the ERC starting grant number 803880 and EpilepsieNL.

WO was supported by the MING foundation.

## Declaration of competing interest

None of the authors have potential conflicts of interest to be disclosed.

## Data Availability

The data that support the findings of this study are being made available in BIDS format on OpenNeuro: https://openneuro.org/datasets/ds005448. The code to analyze the data and generate all figures of this manuscript is available on GitHub: https://github.com/UMCU-EpiLAB/umcuEpi_CCEP_DTI.
