# Supplementary material for: Structural and effective brain connectivity in focal epilepsy
Source: Neuroimage Rep. 2025 Jun 27;5(3):100274. doi: 10.1016/j.ynirp.2025.100274 (PMC12489785; doi:10.1016/j.ynirp.2025.100274)
Supplement: Multimedia component 1 [file mmc1.docx]

# Supplementary material

We used Spearman’s $\rho$ test to calculate the correlation between the node proximity and the degree at patient level for both the structural and effective networks. A node with a low node proximity indicates that this electrode contact has a lot of nearby electrode contacts. As expected, the nodes in de SOZ often had low node proximity. The node proximity showed a significant negative correlation with the degree of the node in the structural connectivity network in 10/13 patients and a significant positive correlation in one ECoG patient (Figure S1). The node proximity showed a significant negative correlation with the degree of the node in the effective connectivity network in 12/13 patients (Figure S2).

The electrode contact areas contained a maximum of 64 grey-white matter voxels closest to each coordinate. Overlapping voxels were assigned to the nearest electrode contact. The histogram of the resulting volumes of the electrode contact areas per patient is shown in Figure S3. We used Spearman’s $\rho$ test to calculate the correlation between the volume of electrode areas and the degree of structural networks. We did not find a consistent correlation (Figure S4).

We did not find a correlation for the betweenness centrality between structural and effective connectivity for most patients (10/13) as shown in Figure S5.


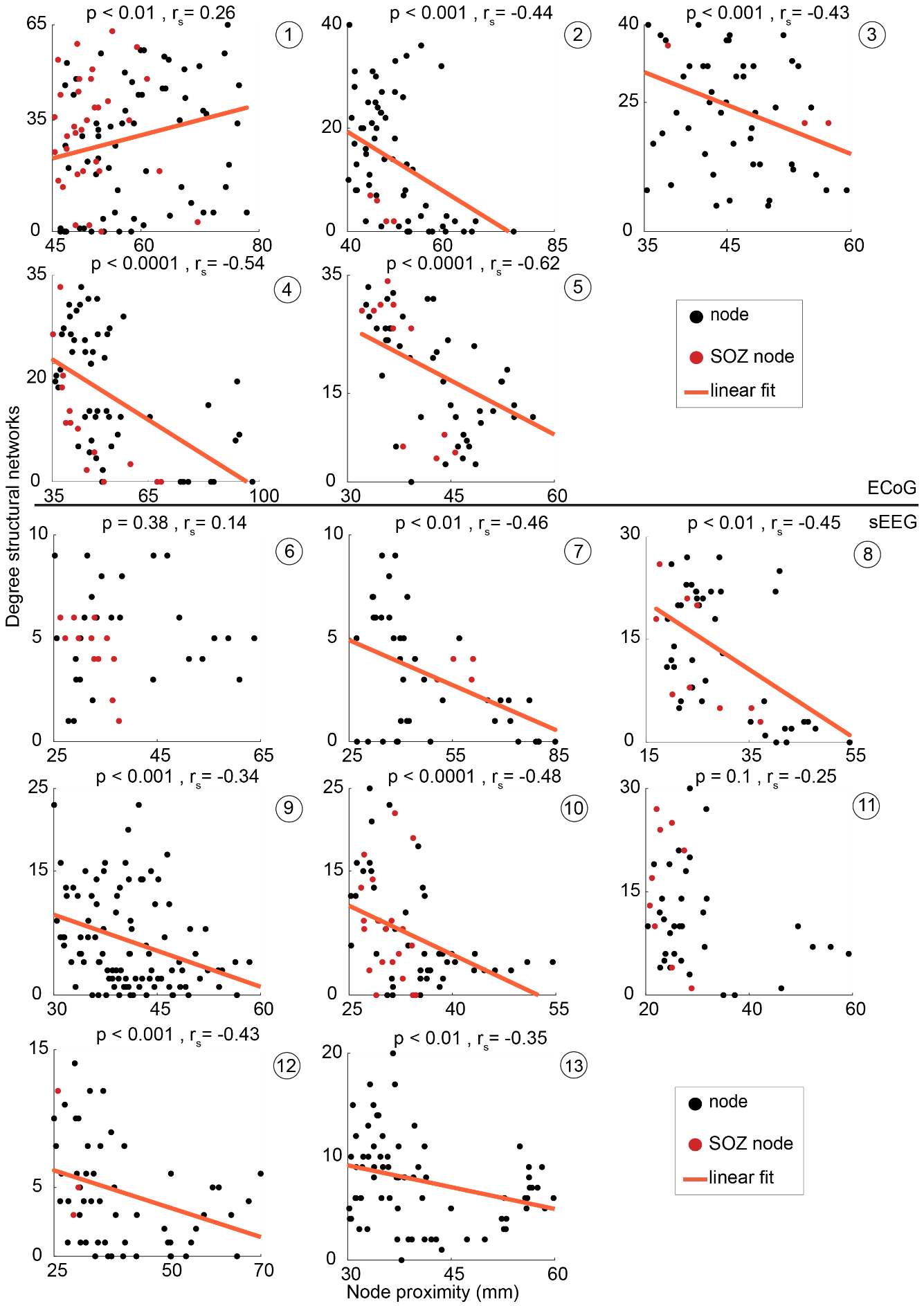
Figure S1: **Correlation between the node proximity and the degree of structural networks.**
In 10/13 patients the node proximity was negatively correlated with the degree of structural networks. Patient 1 showed a significant positive correlation and patients 6 and 11 showed no significant correlation. The orange lines are the best linear fit through the data points. The purple markers indicate the seizure onset zone (SOZ) nodes. In patient 9 and 13 the SOZ was not determined. We corrected for multiple testing with FDR-correction (p<0.05). SOZ = seizure onset zone, $r_{s}$ = correlation coefficient spearman’s ρ test, sEEG = stereo EEG, ECoG = electrocorticogram


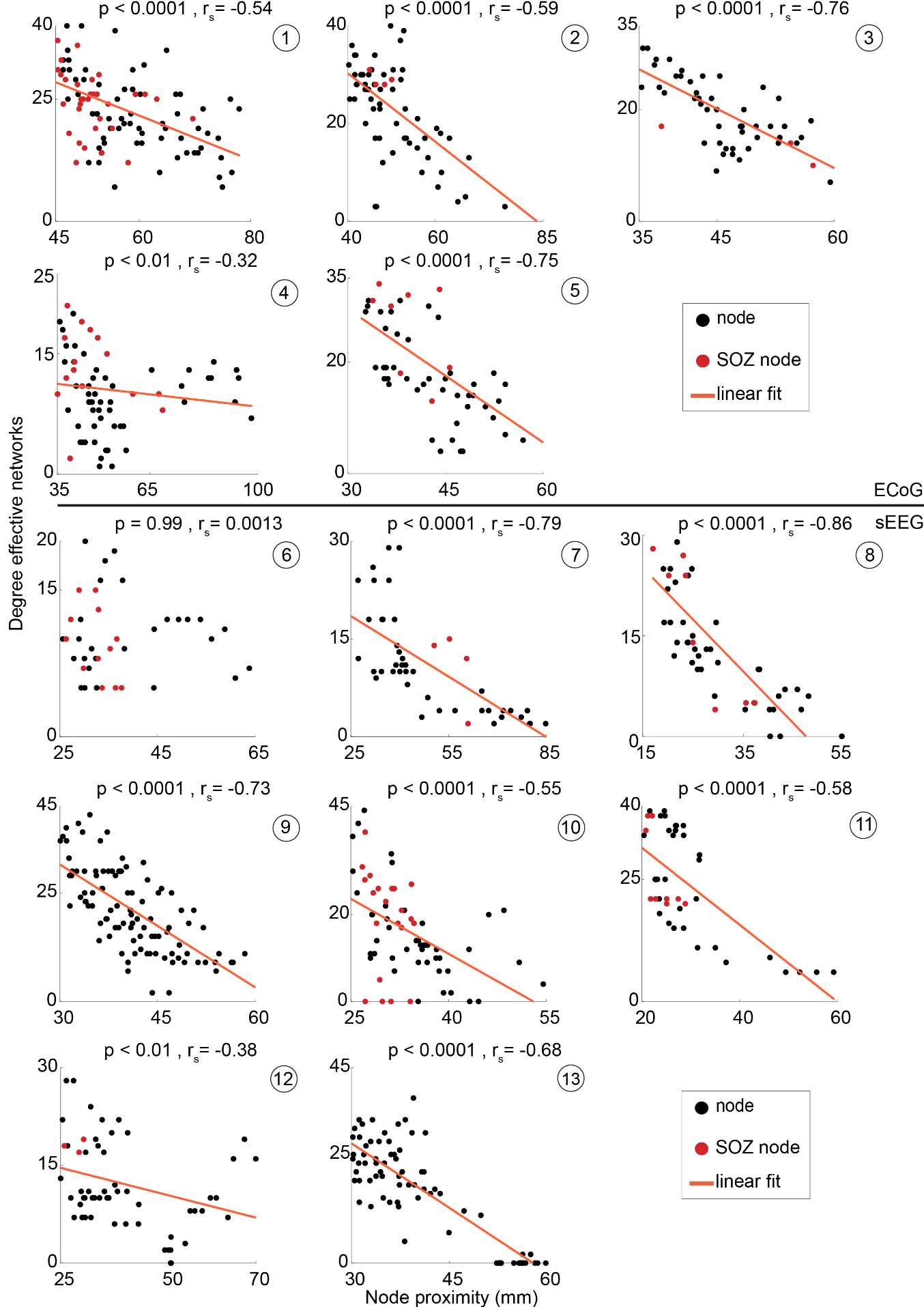


Figure S2: **Correlation between the node proximity and the degree of effective networks.**
In 12/13 patients the node proximity was negatively correlated with the degree of effective networks. Patient 6 showed no significant correlation. The orange lines are the best linear fit through the data points. The purple markers indicate the seizure onset zone (SOZ) nodes. In patient 9 and 13 the SOZ was not determined. We corrected for multiple testing with FDR-correction (p<0.05). SOZ = seizure onset zone, $r_{s}$ = correlation coefficient spearman’s ρ test, sEEG = stereo EEG, ECoG = electrocorticogram


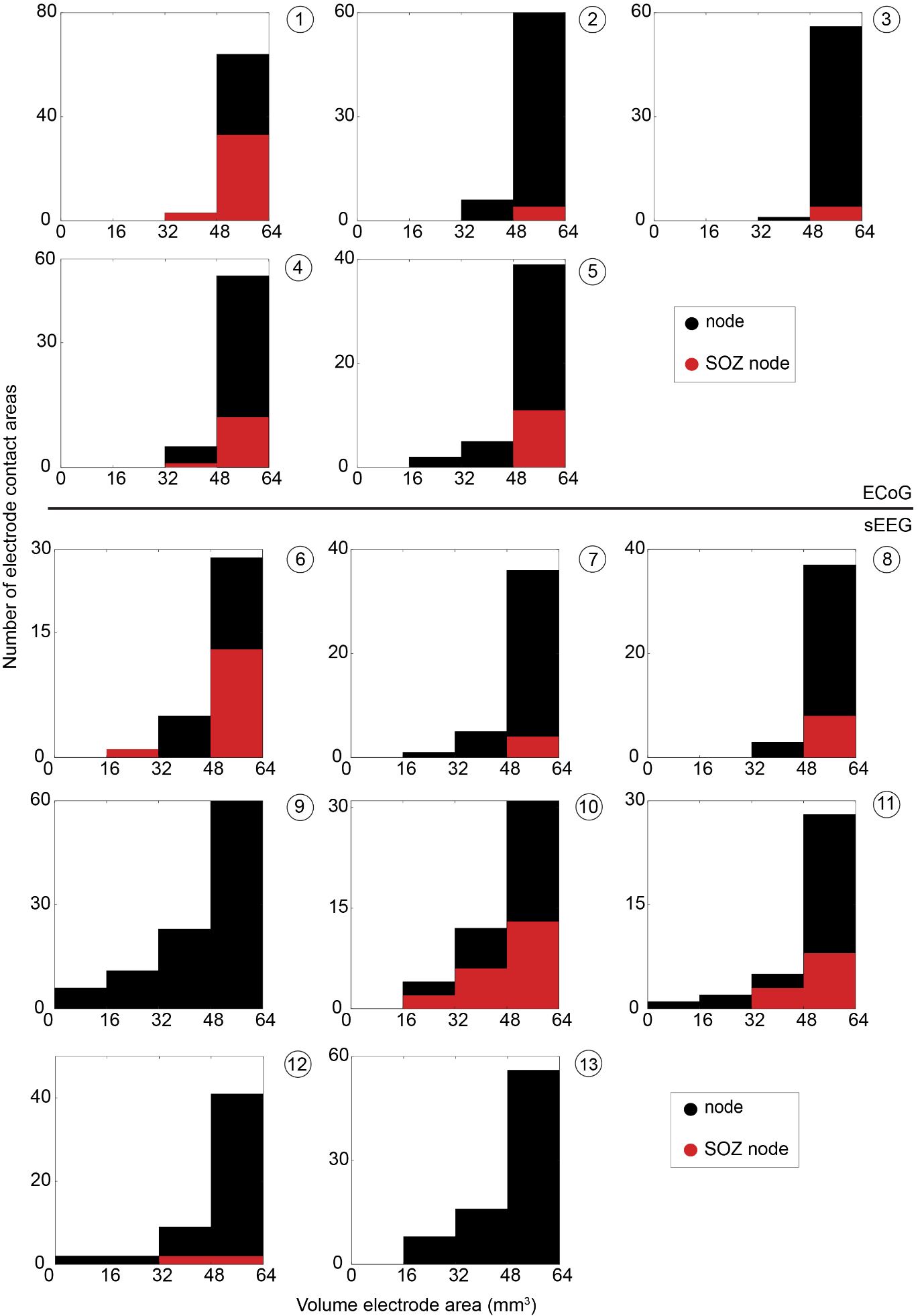


Figure S3: **Histogram of the volume of electrode contact areas.**
Most electrode contact areas had a volume between 48 and 64 mm^3.^ The red bars indicate the electrode areas in the seizure onset zone (SOZ). In patient 9 and 13 the SOZ was not determined. SOZ = seizure onset zone, sEEG = stereo EEG, ECoG = electrocorticography


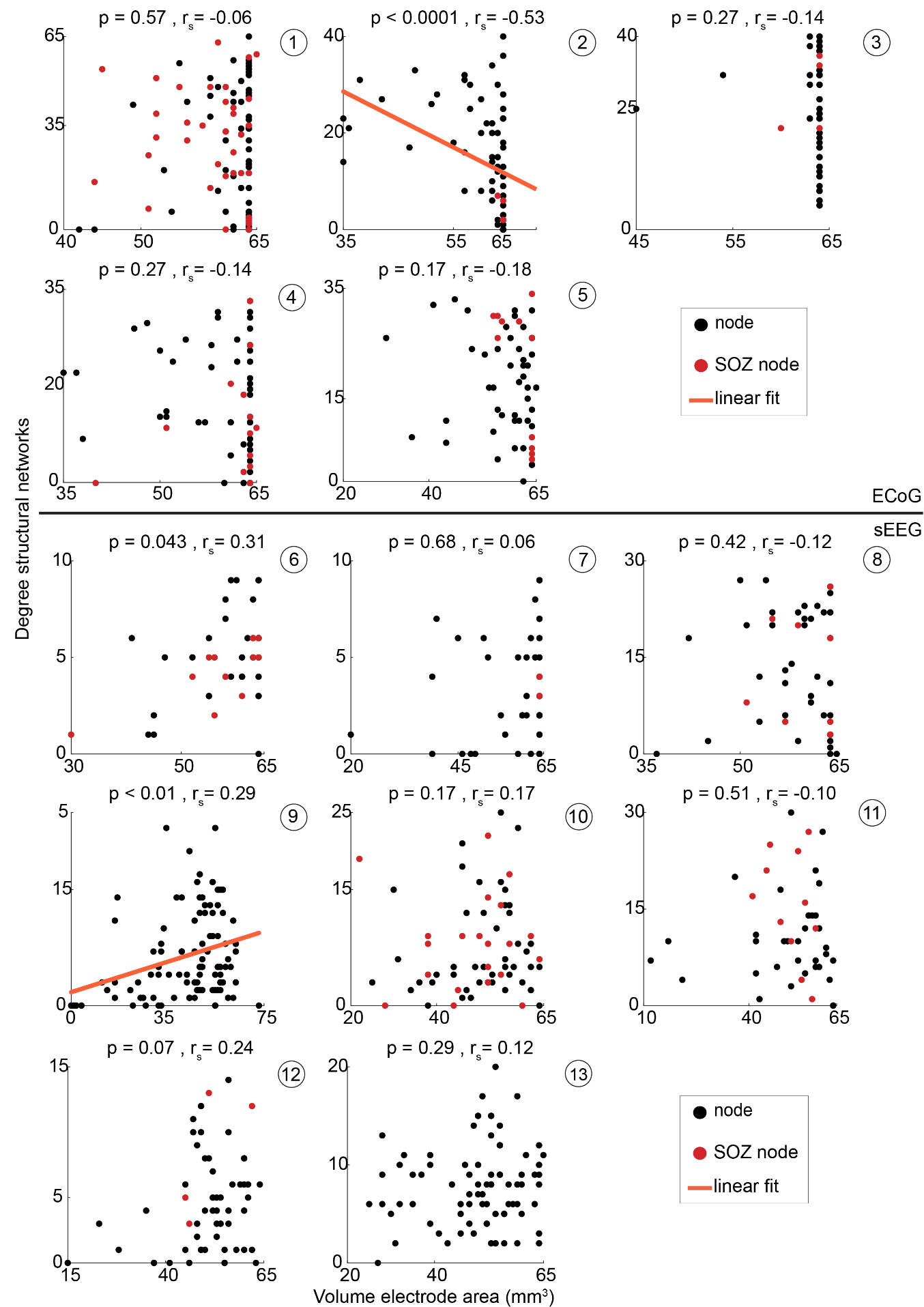


Figure S4: **Correlation between the volume of electrode areas and the degree of structural networks.**
In 11/13 patients the volume of electrode areas was not correlated with the degree of structural networks. Patient 2 showed a significant negative correlation and patients 9 showed a significant positive correlation. The orange lines are the best linear fit through the data points. The purple markers indicate the seizure onset zone (SOZ) nodes. In patient 9 and 13 the SOZ was not determined. We corrected for multiple testing with FDR-correction (p<0.05). SOZ = seizure onset zone, $r_{s}$ = correlation coefficient spearman’s ρ test, sEEG = stereo EEG, ECoG = electrocorticogram


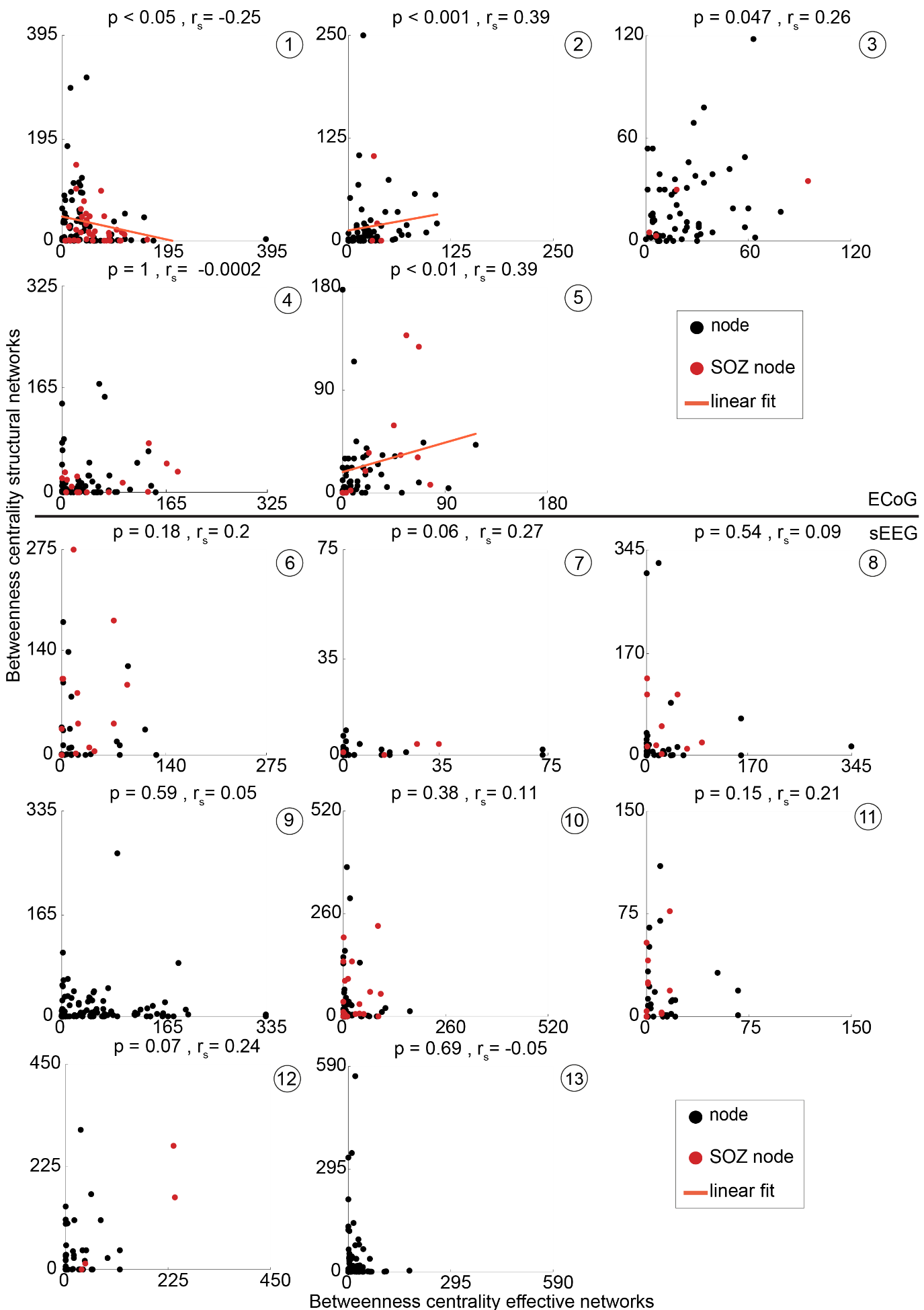
Figure S5: Correlation between the betweenness centrality of structural and effective networks per patient. In 3/5 ECoG patients, the betweenness centrality of the structural networks was positively correlated to the betweenness centrality of the effective networks. The orange lines are the best linear fit through the data points. The purple markers indicate the seizure onset zone (SOZ) nodes. In patient 9 and 13 the SOZ was not determined. We corrected for multiple testing with FDR-correction (p<0.05). SOZ = seizure onset zone, $r_{s}$ = correlation coefficient spearman’s ρ test, sEEG = stereo EEG, ECoG = electrocorticogram.
